# FOXO1 enhances G6PD expression to promote cancer cell antioxidative capacity

**DOI:** 10.1093/jmcb/mjaf021

**Published:** 2025-10-22

**Authors:** Xianhong Zhang, Jie Zhang, Mengmeng Wei, Min Zhao, Xiaoxiong Wang, Yongfeng Hui, Dongdong Yuan, Zijiao Wang, Wei Wu, Peng Jiang, Yujiong Wang, Le Li

**Affiliations:** School of Life Sciences, Ningxia University, Yinchuan 750021, China; Key Laboratory of Ministry of Education for Protection and Utilization of Special Biological Resources in Western China, Ningxia University, Yinchuan 750021, China; School of Life Sciences, Ningxia University, Yinchuan 750021, China; Key Laboratory of Ministry of Education for Protection and Utilization of Special Biological Resources in Western China, Ningxia University, Yinchuan 750021, China; School of Life Sciences, Ningxia University, Yinchuan 750021, China; Key Laboratory of Ministry of Education for Protection and Utilization of Special Biological Resources in Western China, Ningxia University, Yinchuan 750021, China; School of Life Sciences, Ningxia University, Yinchuan 750021, China; Key Laboratory of Ministry of Education for Protection and Utilization of Special Biological Resources in Western China, Ningxia University, Yinchuan 750021, China; General Hospital of Ningxia Medical University, Yinchuan 750021, China; General Hospital of Ningxia Medical University, Yinchuan 750021, China; School of Life Sciences, Ningxia University, Yinchuan 750021, China; Key Laboratory of Ministry of Education for Protection and Utilization of Special Biological Resources in Western China, Ningxia University, Yinchuan 750021, China; School of Life Sciences, Ningxia University, Yinchuan 750021, China; Key Laboratory of Ministry of Education for Protection and Utilization of Special Biological Resources in Western China, Ningxia University, Yinchuan 750021, China; School of Life Sciences, Ningxia University, Yinchuan 750021, China; Key Laboratory of Ministry of Education for Protection and Utilization of Special Biological Resources in Western China, Ningxia University, Yinchuan 750021, China; School of Life Sciences, Tsinghua University, Beijing 100084, China; School of Life Sciences, Ningxia University, Yinchuan 750021, China; Key Laboratory of Ministry of Education for Protection and Utilization of Special Biological Resources in Western China, Ningxia University, Yinchuan 750021, China; School of Life Sciences, Ningxia University, Yinchuan 750021, China; Key Laboratory of Ministry of Education for Protection and Utilization of Special Biological Resources in Western China, Ningxia University, Yinchuan 750021, China

**Keywords:** FOXO1, G6PD, reactive oxygen species, survival, proliferation

## Abstract

Metabolic adaptability, controlled by transcription factors or oncogenes, is critical for the survival of cancer cells. However, the mechanism by which the transcription factor forkhead box protein O1 (FOXO1) regulates the proliferation and survival of malignant tumor cells under high levels of reactive oxygen species (ROS) remains poorly understood. Here, we found that FOXO1 endows cancer cells with the strong antioxidative capacity and rapid proliferation. By upregulating the expression of glucose-6-phosphate dehydrogenase (G6PD), the rate-limiting enzyme in the pentose phosphate pathway, FOXO1 promotes the synthesis of nicotinamide adenine dinucleotide phosphate and ribose 5-phosphate and thus enhances the antioxidative and proliferative capabilities of cancer cells. Induction of G6PD expression in FOXO1-deficient cells mitigates tumor growth inhibition and alleviates ROS level elevation. These results establish a critical role of FOXO1 in the regulation of G6PD during the antioxidative and proliferative processes of cancer cells.

## Introduction

The ‘O’ subclass of the forkhead transcription factor (FOX) family comprises four members, namely FOXO1 (FKHR), FOXO3 (FKHRL1), FOXO4 (AFX), and FOXO6, among which, forkhead box protein O1 (FOXO1) is the most representative member and plays a crucial role in transcriptional regulation. It has been shown that FOXO1 contributes to the development of gastric and epithelial ovarian cancers (Aird and Zhang, 2015), regulates cellular metabolic reprogramming in response to intrinsic or extrinsic stress, as well as controls cancer cell apoptosis through the regulation of target genes (Salih and Brunet, 2008; Hornsveld et al., 2018). Notably, reactive oxygen species (ROS) and other stress stimuli that trigger ROS generation can modulate FOXO1 activity at multiple levels, including phosphorylation, acetylation, and ubiquitination (Yang and Hung, 2009). In turn, the activated FOXO1 can regulate the expression of antioxidant genes and eliminate cellular ROS, thus preventing oxidative damage (Tiwari et al., 2016).

Oxidative stress not only promotes tumorigenesis by increasing the rate of DNA mutation or inducing DNA damage and cell proliferation but also suppresses tumorigenesis by inducing senescence and apoptosis (Wang et al., 2021). Therefore, cancer cells must optimize cellular ROS levels to maintain tumor proliferation. The transcription factor FOXO1 acts as a mediator of oxidative stress activated by cellular stress responses. However, the expression of some antioxidant genes regulated by FOXO1 does not fully explain the effect of FOXO1 on ROS, which is favorable for tumor cell survival. Other antioxidants mediated by FOXO1 may also be involved in the antioxidant defense processes of cancer cells. In addition, it remains unclear how FOXO1 maintains ROS homeostasis while regulating tumor cell proliferation at high ROS levels.

The pentose phosphate pathway (PPP) provides ribose and nicotinamide adenine dinucleotide phosphate (NADPH) to support biosynthesis and antioxidant defense. NADPH is critical for antioxidant responses, generating reduced glutathione (GSH) to protect against protein damage and providing the ultimate reducing power for the function of several other antioxidant systems (Ferri et al., 2005). In addition, PPP itself is directly regulated by multiple mechanisms in response to cellular demand for ROS detoxification and biosynthesis, focusing on the pacemaker of oxidative PPP, glucose-6-phosphate dehydrogenase (G6PD) (Jiang et al., 2013b). G6PD is a key enzyme for the production of NADPH in the PPP. It increases the NADPH pool by stimulating NAD^+^ kinase-mediated NADP^+^ biosynthesis and converting NADP^+^ to NADPH, thereby enhancing antioxidant defenses (Stanton, 2012). Furthermore, the PPP is oncogenically and metabolically regulated by numerous factors, including tumor suppressors, oncoproteins, and intracellular metabolites. Dysregulation of PPP flux dramatically affects cancer cell growth and survival (Jiang et al., 2014).

Here, we report that silencing FOXO1 leads to elevated ROS levels in tumor cells, which reduces tumor cell proliferation and survival under oxidative stress conditions. The specific mechanism is that FOXO1 transcriptionally activates G6PD to enhance the antioxidative capacity and, consequently, the anti-apoptotic and proliferative abilities of tumor cells in response to oxidative stress. This explains how cancer cells rewire their metabolism from glycolysis to the PPP to obtain an adequate supply of the NADPH reductant to detoxify ROS via FOXO1. Meanwhile, the upregulated G6PD expression by FOXO1 can promote PPP progression and further enhance the growth of tumor cells *in vivo* and *in vitro*. These findings indicate that FOXO1 responds to cellular stress in a timely manner by upregulating the expression of G6PD and coordinating the production of NADPH, which maintains redox homeostasis and inhibits ROS accumulation and also endows tumor cells with the ability to proliferate rapidly.

## Results

### FOXO1 promotes G6PD expression

**Figure 1:**
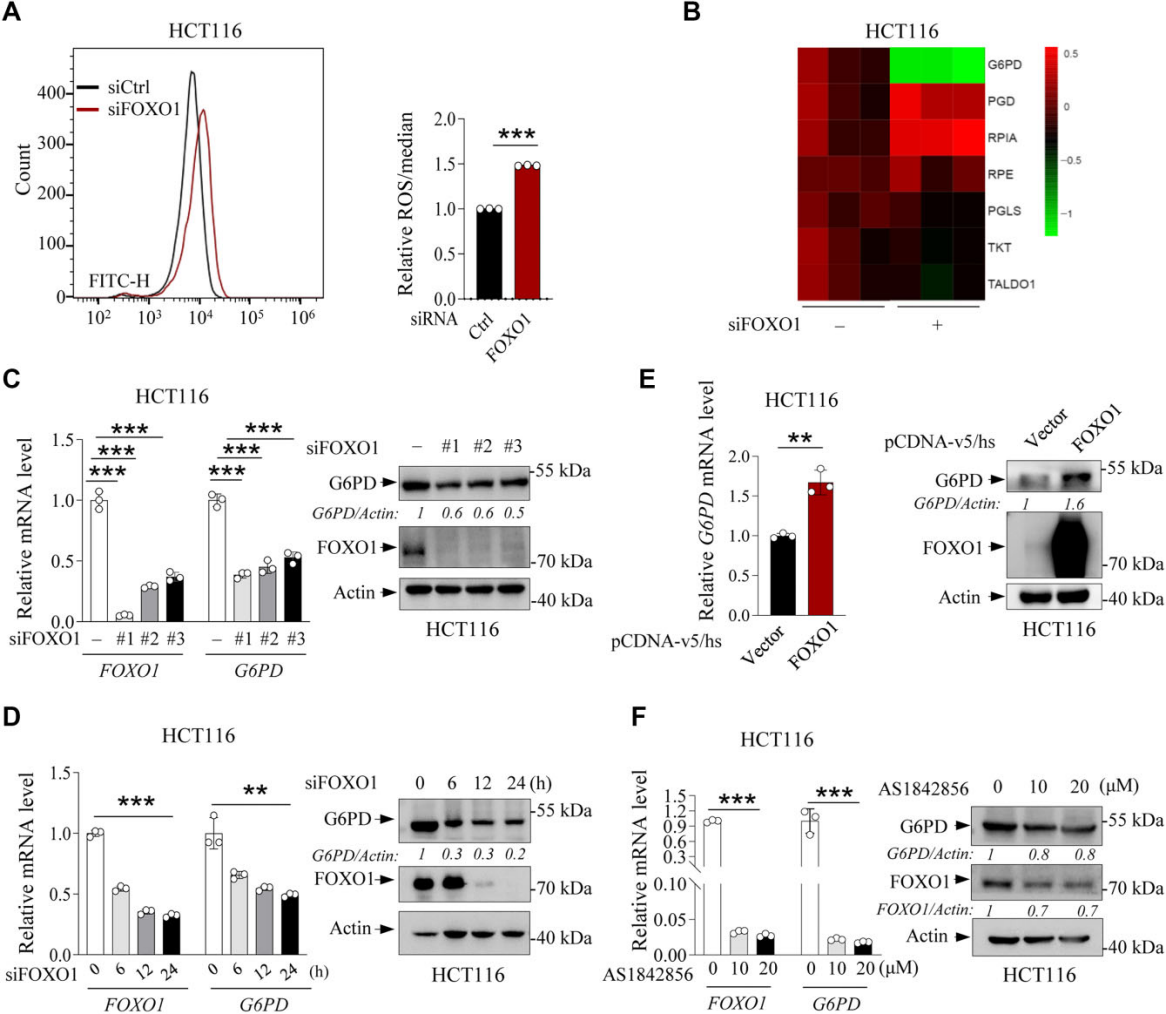
FOXO1 regulates the expression of G6PD. (**A**) ROS levels in HCT116 cells transfected with FOXO1 siRNA or control siRNA. Cells were incubated in 1× PBS containing 10 μM DCFH-DA at 37°C for 30 min in dark. ROS levels were determined by flow cytometry and presented as mean fluorescence intensity values (± SD). (**B**) Heatmap showing the differential expression of known FOXO1 target genes by RNA-seq with HCT116 cells transfected with FOXO1 siRNA or control siRNA. (**C**) HCT116 cells were transfected with control or FOXO1 siRNA as indicated. The mRNA levels of *FOXO1* and *G6PD* were determined by qRT-PCR (left) and representative blots from three independent experiments show the protein levels of G6PD and FOXO1 (right). (**D**) HCT116 cells were transfected with FOXO1 siRNA for various durations and analyzed for mRNA and protein levels of G6PD and FOXO1. (**E**) HCT116 cells were transfected with pCDNA-v5/hs-vector or pCDNA-v5/hs-FOXO1 and analyzed for *G6PD* mRNA levels and protein levels of G6PD and FOXO1. (**F**) HCT116 cells were treated with various doses of AS1842856 and analyzed for mRNA and protein levels of G6PD and FOXO1. Data are presented as mean ± SD (*n* = 3). *P < 0.05, **P < 0.01, ***P < 0.001, unpaired two-tailed *t*-test.

FOXO1 has been reported to play a regulatory role in maintaining redox equilibrium (Klotz et al., 2015). We first compared ROS levels in control and HCT116 (colorectal cancer) cells with FOXO1 knockdown or overexpression. Knockdown of FOXO1 accelerated ROS accumulation and led to significantly higher levels of ROS compared to the control (Figure 1A), while FOXO1overexpression significantly reduced ROS levels (Supplementary Figure S1A), suggesting that FOXO1 could affect ROS levels in tumor cells. Interestingly, among various cancer cell lines examined, HCT116 cells exhibited the highest mRNA and protein levels of FOXO1, while MDA-MB-231 (breast cancer) and H1299 (lung cancer) cells displayed comparatively lower expression (Supplementary Figure S1B). Furthermore, in 4 out of 7 colorectal cancer patients, the mRNA levels of FOXO1 in tumor tissues were much higher than those in the adjacent normal tissues (Supplementary Figure S1C).

To identify the downstream effector of FOXO1, we performed RNA sequencing (RNA-seq) with control and FOXO1-depleted HCT116 cells. Among the PPP-related redox regulators, G6PD exhibited the most remarkable reduction after FOXO1 knockdown (Figure 1B), which was validated by quantitative real-time polymerase chain reaction (qRT-PCR) (Supplementary Figure S1D). qRT-PCR and western blot results demonstrated that FOXO1 knockdown led to a concomitant reduction in G6PD mRNA and protein levels (Figure 1C and D; Supplementary Figure S1E), while overexpression of FOXO1 led to a significant increase in the mRNA and protein levels of G6PD (Figure 1E), confirming that FOXO1 upregulates G6PD expression. Previous studies indicated that p53 interacts with FOXOs and G6PD (Wang et al., 2008; Zhao et al., 2010; Jiang et al., 2011). Here, we observed that FOXO1 knockdown similarly reduced G6PD expression in HCT116 p53^+/+^ and HCT116 p53^−/−^ cells (Supplementary Figure S1F), suggesting that FOXO1 upregulates the expression of G6PD independently of p53. Furthermore, when HCT116 cells were treated with different concentrations of the FOXO1 transcriptional inhibitor AS1842856 (Li et al., 2020; Zhang et al., 2023), G6PD protein levels also decreased (Figure 1F). However, the stability of G6PD protein was not affected by FOXO1 depletion, as shown by cycloheximide (CHX) chase assays in HCT116 cells (Supplementary Figure S1G). Collectively, these results suggest that FOXO1 transcriptionally activates G6PD, which is independent of p53.

### Regulation of G6PD expression by FOXO1 under stressed conditions

**Figure 2:**
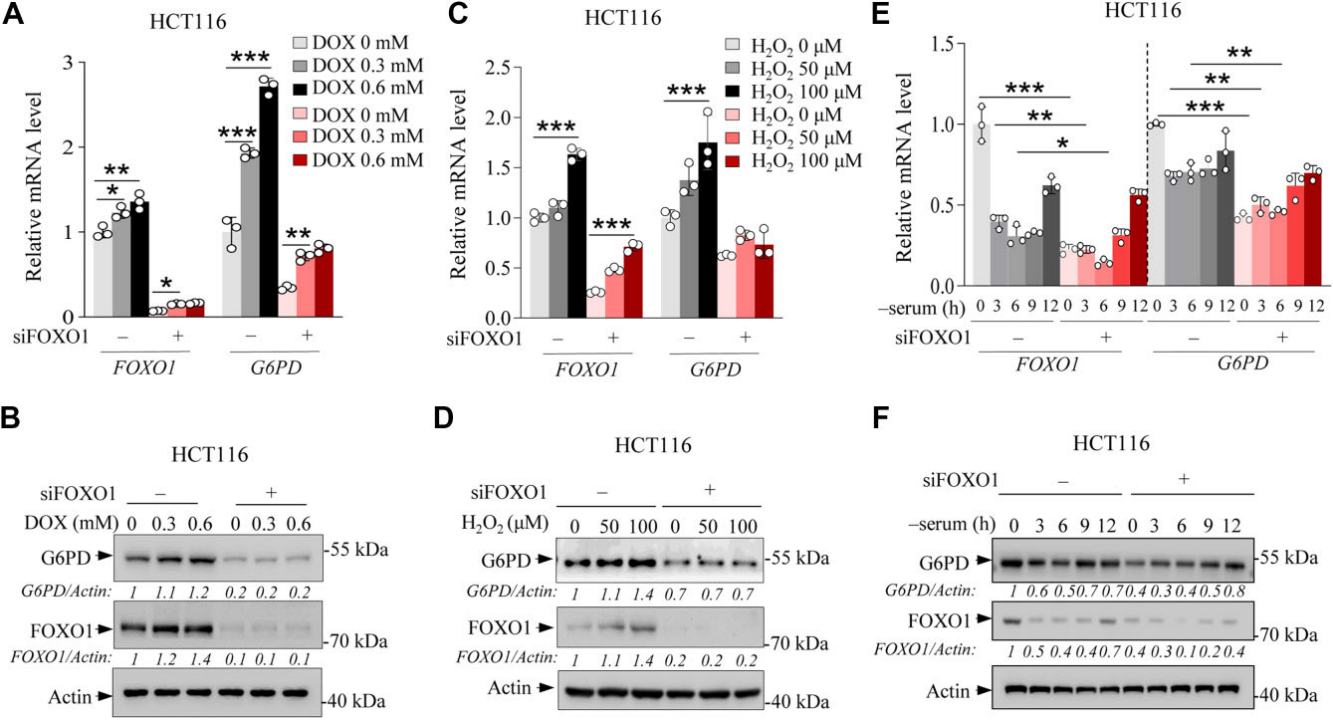
FOXO1 regulates G6PD under stressed conditions. (**A** and **B**) HCT116 cells were transfected with control siRNA or FOXO1 siRNA. After 24 h, the cells were treated with various doses of DOX for 24 h and then analyzed for mRNA and protein levels of G6PD and FOXO1. (**C** and **D**) HCT116 cells were transfected with control siRNA or FOXO1 siRNA. After 24 h, the cells were treated with various doses of H_2_O_2_ for 12 h and then analyzed for mRNA protein levels of G6PD and FOXO1. (**E** and **F**) HCT116 cells were transfected with control siRNA or FOXO1 siRNA and cultured in complete medium for 24 h. Then, the cells were incubated in serum-free medium for various durations and analyzed for mRNA and protein levels of G6PD and FOXO1. Data are representative of three independent experiments and presented as mean ± SD (*n* = 3). *P < 0.05, **P < 0.01, ***P < 0.001, unpaired two-tailed *t*-test.

FOXO1 plays a critical regulatory role under cellular stress conditions. It has been reported that FOXO1 is upregulated during DNA damage (Ju et al., 2014). In our study, treatment with doxorubicin (DOX) led to the concentration-dependent increase in the mRNA and protein levels of FOXO1, and the expression levels of G6PD increased in a FOXO1-dependent manner (Figure 2A and B). It has also been shown that H_2_O_2_-mediated ROS generation activates the AKT/FOXO1 pathway (Li et al., 2020) and H_2_O_2_ regulates FOXO1 activity showing a pronounced dose-dependent characteristic (Jose et al., 2024). We found that the mRNA and protein levels of FOXO1 increased with the accumulation of H_2_O_2_ and G6PD levels increased in a FOXO1-dependent manner (Figure 2C and D). In addition, when HCT116 cells were cultured in serum-deprived medium, G6PD mRNA and protein levels declined rapidly initially (0–6 h) and partially recovered later (∼9–12 h), highly correlated with FOXO1 mRNA and protein levels (Figure 2E and F). Notably, depletion of FOXO1 not only reduced the basal levels of G6PD but also essentially eliminated the fluctuation in G6PD expression (Figure 2E and F). These results indicate that FOXO1 controls G6PD expression in response to DNA damage, ROS accumulation, and growth factor withdrawal.

### G6PD is a transcriptional target gene for FOXO1

**Figure 3:**
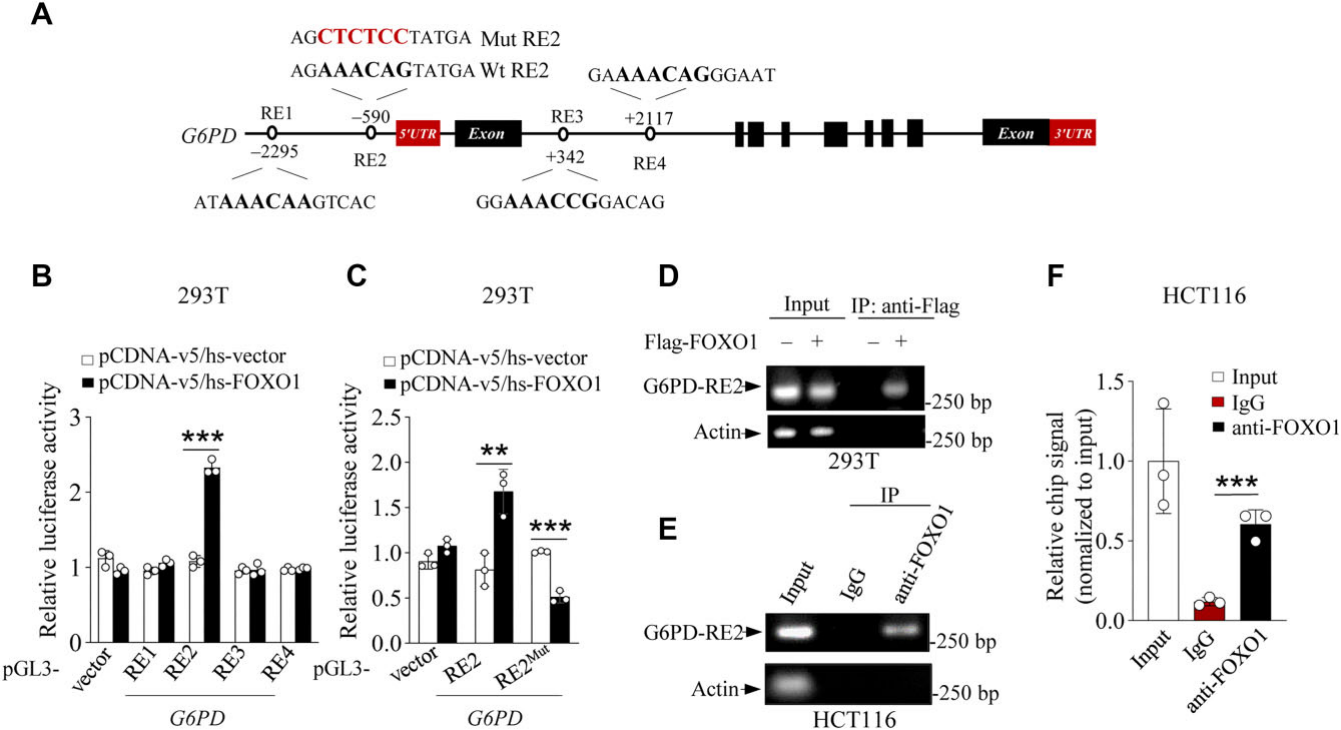
G6PD is a target gene for FOXO1. (**A**) Diagram of the human G6PD genomic structure, showing the sequences of four potential FOXO1 protein REs and the corresponding mutant RE2. (**B** and **C**) Luciferase constructs containing different RE sequences as indicated were transfected into 293T cells together with Flag-FOXO1 or vector control. The pRL-CMV vector was used as an internal transfection control. (**D**) 293T cells transfected with vector control or Flag-FOXO1 were analyzed by ChIP assay using normal rabbit IgG and anti-Flag antibodies, followed by PCR for the bound DNA. (**E**) HCT116 cells were analyzed by ChIP assay using normal rabbit IgG and anti-FOXO1 antibodies, followed by PCR. (**F**) qChIP assay validating G6PD as a direct target of FOXO1. Chromatin was enriched by anti-FOXO1 or anti-rabbit-IgG antibodies. Actin was used as an internal reference gene. Data are presented as mean ± SD (*n* = 3). *P < 0.05, **P < 0.01, ***P < 0.001, unpaired two-tailed *t*-test.

To examine whether FOXO1 is a transcriptional activator of G6PD, we analyzed the human *G6PD* gene sequence for potential FOXO1 protein response elements (REs), which share the consensus sequence of 5′-AAACAG-3. We identified RE1 and RE2 in the 5′ flanking regions and RE3 and RE4 in the first intron (Figure 3A). By cloning the genomic fragment encompassing each RE into the promoter region of a firefly luciferase reporter plasmid, we found that FOXO1 induced luciferase expression driven by RE2 but not by RE1, RE3, or RE4 (Figure 3B). Furthermore, FOXO1 failed to induce luciferase expression driven by mutant RE2 (Mut RE2) in which the six conserved nucleotides were altered (Figure 3C). Chromatin immunoprecipitation (ChIP) assays showed that both Flag-FOXO1 and endogenous FOXO1 associated with the RE2 region of G6PD (Figure 3D and E). The FOXO1 occupancy of this binding site within the G6PD promoter was confirmed by qChIP analysis (Figure 3F). These data indicate that FOXO1 stimulates G6PD expression by binding to RE2.

### FOXO1 regulates NADPH homeostasis

**Figure 4:**
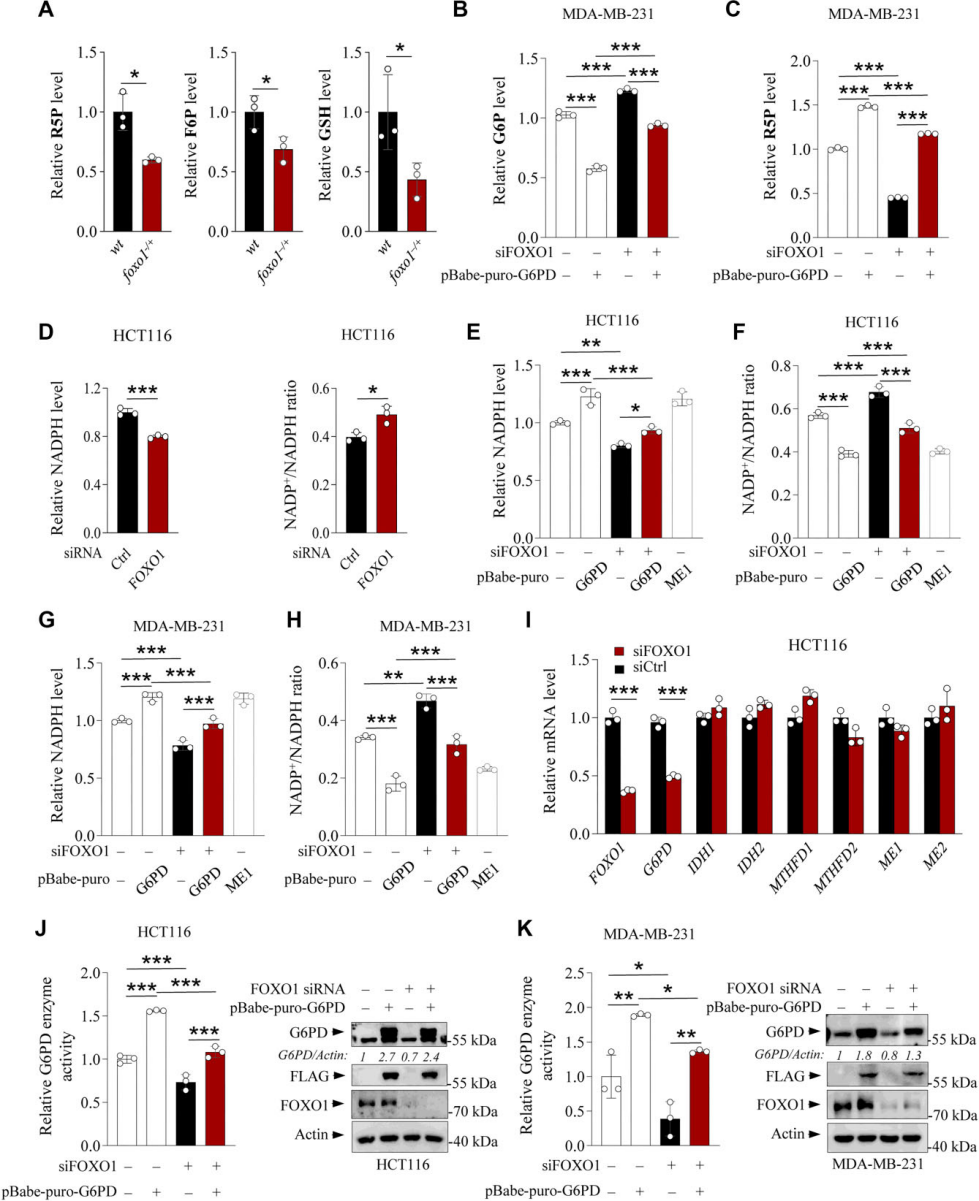
FOXO1 regulates NADPH homeostasis and G6PD activity. (**A**) Serum G6P, R5P, and GSH levels in wild-type (*wt*) and *foxo1*-knockout (*foxo1^−^^/+^*) mice were determined by LC–MS. (**B** and **C**) G6P and R5P levels in MDA-MB-231 cells stably overexpressing G6PD or vector control in the presence or absence of FOXO1 siRNA. (**D**) NADPH levels and NADP^+^/NADPH ratios in HCT116 cells transfected with control or FOXO1 siRNA. (**E** and **F**) NADPH levels and NADP^+^/NADPH ratios in HCT116 cells overexpressing pBabe-puro-G6PD or vector control in the presence or absence of FOXO1 siRNA. Overexpression of ME1 was employed as the positive control. (**G** and **H**) NADPH levels and NADP^+^/NADPH ratios in MDA-MB-231 cells overexpressing pBabe-puro-G6PD or vector control in the presence or absence of FOXO1 siRNA. Overexpression of ME1 was employed as the positive control. (**I**) HCT116 cells were transfected with control siRNA or FOXO1 siRNA. After 48 h, *FOXO1, G6PD, IDH1, IDH2, MTHFD1, MTHFD2, ME1*, and *ME2* mRNA levels were analyzed by qRT-PCR (*n* = 3). (**J** and **K**) G6PD activities and G6PD and FOXO1 protein levels in HCT116 and MDA-MB-231 cells overexpressing pBabe-puro-G6PD or vector control in the presence or absence of FOXO1 siRNA. Data are presented as mean ± SD (*n* = 3). *P < 0.05, **P < 0.01, ***P < 0.001, unpaired two-tailed *t*-test (**A, D, I**) or two-way ANOVA followed by Tukey's multiple-comparison test (**B, C, E**–**H, J, K**).

G6PD is a key enzyme in the PPP, which mediates the production of the downstream metabolite NADPH (Jiang et al., 2014). To investigate whether FOXO1 activates G6PD to promote the PPP, we measured the PPP metabolite levels in the serum of wild-type and *foxo1*-knockout (*foxo1^−^^/+^*) mice. The levels of GSH, ribose 5-phosphate (R5P), and fructose-6-phosphate (F6P) were all significantly lower in *foxo1*-knockout mice (Figure 4A; Supplementary Figure S1H). Furthermore, in MDA-MB-231 cells, knockdown of FOXO1 led to the increased level of G6P and decreased level of R5P; stable overexpression of G6PD rescued the levels of both G6P and R5P (Figure 4B and C). Consistently, in HCT116 cells, G6PD overexpression restored G6P and R5P levels after FOXO1 knockdown (Supplementary Figure S1I and J). These results indicate that FOXO1 might promote the PPP via G6PD.

Next, we investigated whether FOXO1 regulates NADPH biosynthesis via G6PD. Silencing FOXO1 significantly decreases the NADPH level but increases the NADP^+^/NADPH ratio, which can be reversed by overexpressing pBabe-puro-G6PD in FOXO1-silenced HCT116 or MDA-MB-231 cells (Figure 4D–H). NADPH homeostasis is mainly regulated by metabolic pathways, such as the PPP, and metabolic enzymes, including the folate-mediated one-carbon metabolism enzyme methylenetetrahydrofolate dehydrogenase (MTHFD2), malic enzymes (ME1 and ME2), and cytosolic or mitochondrial NADP-dependent isocitrate dehydrogenases (IDH1 and IDH2) (Ju et al., 2020; Rather et al., 2021). Knockdown of FOXO1 significantly downregulated the expression of G6PD but had a minimal effect on other NADPH-producing metabolic enzymes (Figure 4I), suggesting that FOXO1 maintains cellular NADPH homeostasis by stimulating G6PD expression. In addition, FOXO1 knockdown significantly reduced G6PD enzyme activity, while FOXO1 overexpression enhanced the enzyme activity in HCT116 cells (Supplementary Figure S1K). The reduced protein expression and enzyme activity of G6PD in FOXO1-silenced MDA-MB-231 and HCT116 cells were both restored by overexpression of pBabe-puro-G6PD (Figure 4J and K). Collectively, these results indicate that FOXO1 stimulates G6PD expression and its enzyme activity to promote PPP progression and the production of the downstream metabolite NADPH.

### FOXO1 enhances cellular antioxidative capacity via G6PD

**Figure 5:**
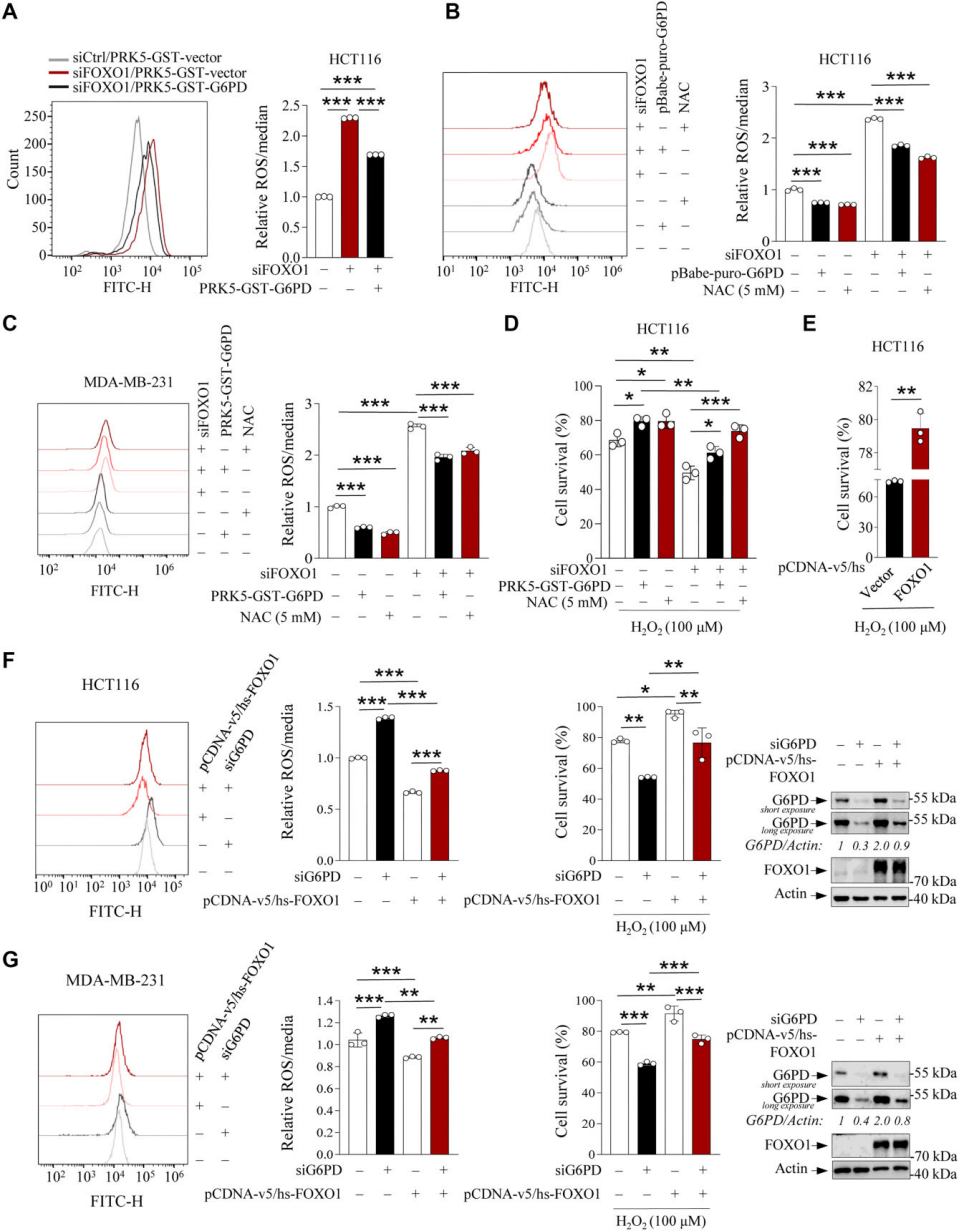
FOXO1 promotes G6PD expression to enhance cellular antioxidative capacity. (**A**) ROS levels in HCT116 cells overexpressing G6PD or vector control in the presence or absence of FOXO1 siRNA were determined by flow cytometry. (**B** and **C**) ROS levels in HCT116 and MDA-MB-231 cells overexpressing G6PD or vector control in the presence or absence of FOXO1 siRNA or NAC. (**D**) HCT116 cells were transfected with vector control or PRK5-GST-G6PD in the presence or absence of FOXO1 siRNA or NAC and treated with 100 μM H_2_O_2_ for 24 h. Cell survival was assayed by trypan blue stain. (**E**) Cell survival analysis of HCT116 cells transfected with pCDNA-v5/hs-vector or pCDNA-v5/hs-FOXO1 and treated with 100 μM H_2_O_2_ for 24 h. (**F** and **G**) ROS levels, cell survival rates, G6PD and FOXO1 protein levels in HCT116 and MDA-MB-231 cells overexpressing pCDNA-v5/hs-FOXO1 or pCDNA-v5/hs-vector in the presence or absence of G6PD siRNA. Data are presented as mean ± SD (*n* = 3). *P < 0.05, **P < 0.01, ***P < 0.001, unpaired two-tailed *t*-test (**A, E**) or two-way ANOVA followed by Tukey's multiple-comparison test (**B**–**D, F, G**).

G6PD plays a vital role in the antioxidant metabolism by producing NADPH, which regenerates glutathione (Pandolfi et al., 1995). Previous studies have reported that the reduced G6PD levels could increase oxidative stress in cancer cells (Du et al., 2013). Here, we found that FOXO1 knockdown in HCT116 and MDA-MB-231 cells resulted in higher ROS levels, which were reduced by overexpressing G6PD or adding the ROS scavenger *N*-acetyl-L-cysteine (NAC) (Figure 5A–C; Supplementary Figure S2A), suggesting that FOXO1 can sufficiently reduce cellular ROS levels by regulating G6PD expression.

Next, we examined the effect of FOXO1 on cellular capacity to withstand oxidative stress. FOXO1 knockdown significantly reduced the survival rate of HCT116 cells treated with H_2_O_2_, which can be partially rescued by overexpressing G6PD or adding NAC in these cells (Figure 5D; Supplementary Figure S2B and C), suggesting that FOXO1 can enhance the resistance to H_2_O_2_ by regulating G6PD expression. Indeed, forced expression of FOXO1 also significantly enhanced the survival rate of HCT116 cells treated with H_2_O_2_ (Figure 5E). G6PD knockdown in HCT116 and MDA-MB-231 cells significantly increased cellular ROS content and susceptibility to H_2_O_2_ treatment, while forced expression of FOXO1 significantly increased the survival rate of G6PD-deficient cells (Figure 5F and G), suggesting that FOXO1 enhances cellular antioxidative capacity via G6PD.

### G6PD upregulation contributes to FOXO1-mediated antioxidative response

**Figure 6:**
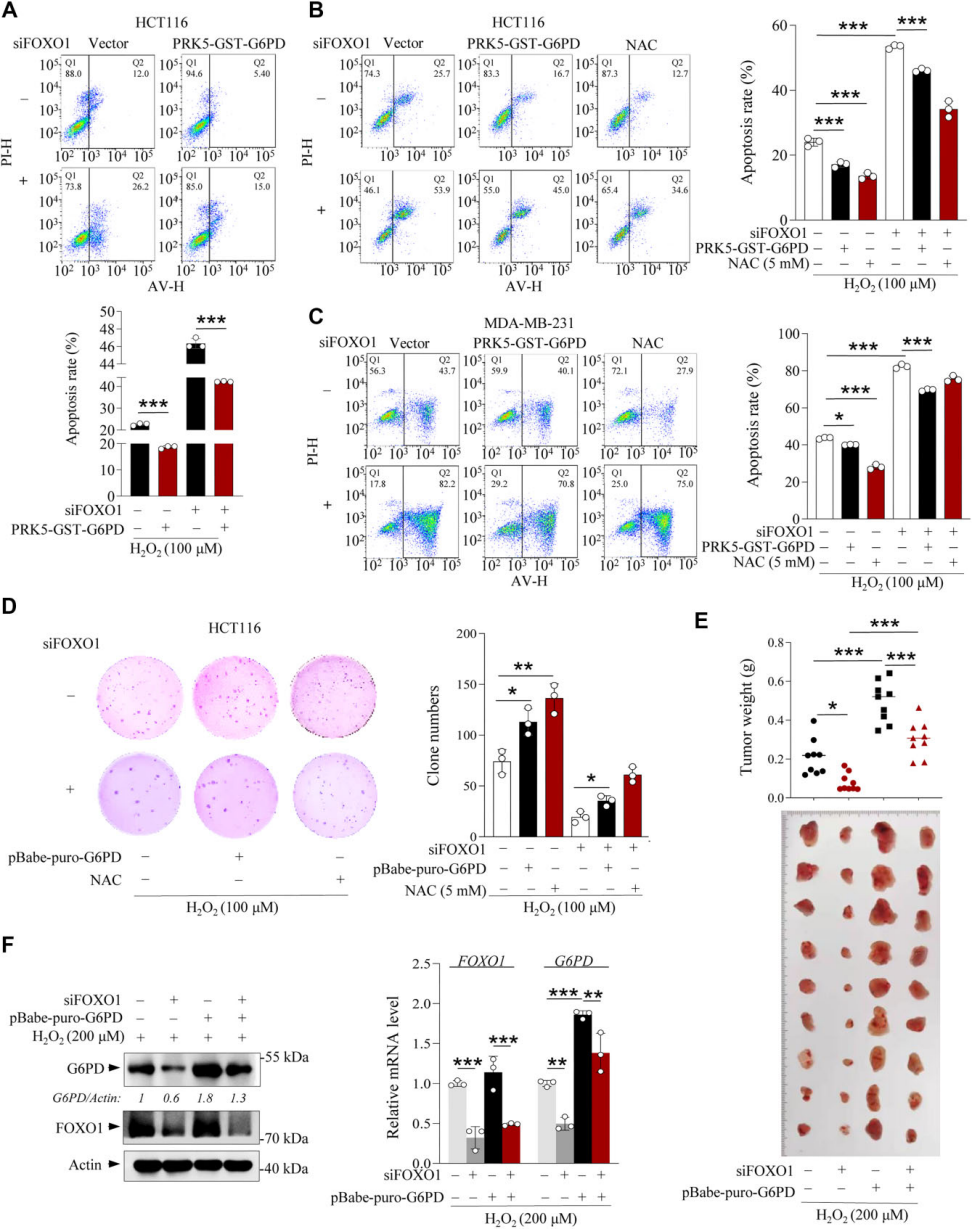
G6PD is involved in FOXO1-mediated antioxidative response under oxidative stress. (**A**) HCT116 cells overexpressing PRK5-GST-G6PD or vector control in the presence or absence of FOXO1 siRNA were treated with 100 μM H_2_O_2_ for 24 h. Cell death was analyzed by flow cytometry after Annexin V-FITC (AV)/propidum iodide (PI) staining. (**B**) Cell death analysis of HCT116 cells overexpressing PRK5-GST-G6PD or vector control in the presence or absence of FOXO1 siRNA or NAC and treated with 100 μM H_2_O_2_ for 24 h. (**C**) Cell death analysis of MDA-MB-231 cells overexpressing PRK5-GST-G6PD or vector control in the presence or absence of FOXO1 siRNA or NAC and treated with 100 μM H_2_O_2_ for 36 h. (**D**) HCT116 cells overexpressing pBabe-puro-G6PD or vector control in the presence or absence of FOXO1 siRNA or NAC were treated with 100 μM H_2_O_2_. Cell proliferation was analyzed by a soft agar colony formation experiment. Representative images showing cells stained with crystal violet on Day 14. (**E**) Average weights and images of xenograft tumors (4 weeks, *n* = 9) generated by FOXO1-silenced MDA-MB-231 cells stably expressing G6PD or vector control as indicated. (**F**) mRNA and protein levels of G6PD and FOXO1 in xenograft tumor samples in **E**. Data are presented as mean ± SD (*n* = 3, except in **E**). *P < 0.05, **P < 0.01, ***P < 0.001, two-way ANOVA followed by Tukey's multiple-comparison test.

To examine whether G6PD is essential for FOXO1-mediated cellular antiapoptotic response to oxidative stress, we performed flow cytometry in the presence or absence of FOXO1 under oxidative stress conditions. Specifically, the loss of FOXO1 increased apoptosis in HCT116 and MDA-MB-231 cells treated with H_2_O_2_ (100 μM), which was partially reversed by overexpressing G6PD or adding NAC (Figure 6A–C; Supplementary Figure S3A), while overexpression of FOXO1 alone significantly decreased tumor cell apoptosis (Supplementary Figure S3B). Next, we examined whether G6PD contributes to FOXO1-driven cell proliferation under oxidative stress. As expected, overexpression of G6PD restored the cell number in FOXO1-silenced HCT116 cells treated with H_2_O_2_ (Supplementary Figure S3C). Notably, overexpression of FOXO1 alone showed a significant proliferation advantage (Supplementary Figure S3D). Consistently, stable overexpression of G6PD or NAC addition rescued the colony-forming ability of FOXO1-deficient cells (Figure 6D). To investigate the roles of FOXO1 and G6PD in tumor growth at high ROS levels *in vivo*, we injected control and G6PD-overexpressing MDA-MB-231 cells, with or without FOXO1 knockdown, into immunocompromised mice and treated with H_2_O_2_ (200 μM) via intratumoral injection every week from Week 2 to Week 4. As shown in Figure 6E and F, overexpression of G6PD alone in control MDA-MB-231 cells led to stronger tumorigenicity at high ROS levels, while knockdown of FOXO1 significantly reduced tumor growth, but knockdown of FOXO1 had a minimal effect on tumor growth of G6PD-overexpressing cells, suggesting that the overexpressed G6PD is strongly tumorigenic at high ROS levels. It has been reported that the high level of G6PD expression indicates the dismal clinical results of various cancer patients and plays a key role in tumorigenesis and chemical resistance (Yang et al., 2019). Indeed, a survey of Kaplan–Meier Plotter database revealed that the elevated expression of G6PD or FOXO1 significantly correlated with poor prognosis in patients (Supplementary Figure S4). Collectively, these results indicate that FOXO1-stimulated G6PD expression in cancer cells can promote their antioxidative capacity, thus reducing apoptosis, increasing proliferation, and enhancing tumor growth under oxidative stress conditions.

## Discussion

G6PD is a critical antioxidant enzyme that facilitates nucleotide biosynthesis through R5P production while maintaining redox homeostasis via NADPH regeneration and GSH recycling (Yang et al., 2021). Overactive G6PD is associated with the transformation, metastasis, and resistance to therapy in multiple cancer types (Riganti et al., 2012). Previous studies have indicated that the expression of G6PD is regulated by the pre-eminent tumor suppressor p53 and its structural homolog TAp73 (Jiang et al., 2011; Du et al., 2013). However, the mechanistic basis for its role in regulating tumor cell proliferation and intracellular ROS accumulation remains incomplete. This study identified FOXO1 as a novel transcriptional activator of G6PD. The dose-dependent regulation of G6PD expression by FOXO1 might represent an important mechanism through which malignant proliferating cells acquire antioxidative capability, underscoring the significance of this transcriptional axis in tumorigenesis.

Metabolic reprogramming enables tumor cells to sustain macromolecular biosynthesis while mitigating oxidative damage (Vander Heiden et al., 2009; Dang, 2012). As the rate-limiting enzyme in the PPP, G6PD critically regulates NADPH production—the principal reducing equivalent countering oxidative stress in malignancies (Filosa et al., 2003). While oncogenic K-Ras and tumor-suppressive p53 modulate NADPH through malic enzyme regulation (Jiang et al., 2013a; Son et al., 2013), our study showed that silencing FOXO1 increased ROS levels and decreased NADPH levels in cancer cells. Furthermore, exogenous NAC supplementation restricted ROS levels in FOXO1-silenced cells to a level akin to G6PD overexpression. These results suggest that FOXO1 contributes to the maintenance of intracellular ROS homeostasis in cancer cells, partially through the modulation of NADPH production mediated by G6PD. Notably, oxidative challenge with H_2_O_2_ upregulated both FOXO1 and G6PD expression levels, reflecting an adaptive feedback mechanism where hyperoxidized peroxiredoxins relieve FOXO1 inhibition under severe oxidative stress (Yang and Hung, 2009; Jose et al., 2024). Exogenous H_2_O_2_ treatment triggers a sharp ROS surge in tumor cells. To counteract this oxidative stress and sustain proliferation, tumor cells upregulate FOXO1 expression to reduce intracellular ROS levels. Moreover, the exogenous addition of DOX could lead to FOXO1 transcriptional upregulation of G6PD expression in response to DNA damage, verifying that G6PD is the transcriptional activation substrate of FOXO1 and FOXO1 responds to cellular stress by regulating G6PD expression.

FOXO1 transcriptionally regulates target genes involved in tumor cell apoptosis and proliferation (Matsuzaki et al., 2016). In this study, silencing FOXO1 increased tumor cell apoptosis under oxidative stress, while overexpressing FOXO1 improved the survival of cancer cells at high ROS levels, highlighting the role of FOXO1 in bolstering tumor cell antioxidative capacity and survival. It has been reported that G6PD inhibition could induce apoptosis and suppress cellular proliferation (Zhang et al., 2010), particularly in cells undergoing oxidative stress (Lin et al., 2010). Here, we observed that stable overexpression of G6PD rescued the survival of FOXO1-silenced cells under oxidative stress. This emphasizes that FOXO1 induces the resistance to oxidative stress in cancer cells via transcriptional activation of G6PD. Importantly, the elevated G6PD activity correlates with poor clinical outcomes and contributes to tumor progression and therapy resistance across multiple cancer types (Wang et al., 2020; Zhang et al., 2021).

In summary, our findings identify the unanticipated roles of FOXO1 in regulating the PPP. As an oxidative stress-sensitive transcription factor, FOXO1 binds to RE2 and activates G6PD expression, driving the PPP to produce more NADPH. This enhances the antioxidative capacity of cancer cells, ultimately promoting tumor growth. The significant correlation between high expression of G6PD and FOXO1 and poor prognosis in patients provides profound insights into the clinical implications of our molecular observations. Further elucidation of the molecular mechanisms underlying this process may lead to the development of novel therapeutic approaches targeting cancer metabolism.

## Materials and methods

### Mice

Specific pathogen-free-grade athymic-Balb/c nude male mice (4-week-old) were purchased from the GemPharmatech LLC. All animals were housed in the animal center and received humane care according to the Guidelines for the Management and Use of Laboratory Animals (Ministry of Science and Technology of China, 2006). Animal experiments were conducted following the China Physiological Society's Guiding Principles in the Care and Use of Animals, with the approval of the Animal Care Committee of Ningxia Medical University (No. IACUC-NYLAC-2022-105). The animals were raised in a conventional facility (12-h/12-h light/dark cycle, 23°C ± 1°C, 45% ± 5% relative humidity, ad libitum access to distilled water and commercial rodent food).

For the H_2_O_2_ treatment group, MDA-MB-231 cells were injected subcutaneously into the flanks of 4-week-old athymic Balb/c male mice (9 mice/group). One week later, when the tumors reached a volume of 100 mm^3^, H_2_O_2_ (200 μM) was administered by intratumoral injection every week for 3 weeks. Subsequently, the mice were sacrificed and tumor growth was evaluated at Week 4 post-injection. FOXO1-floxed mice were generated by Shanghai Model Organisms Center using the CRISPR–Cas9 system.

### Cell culture

HCT116, U87, H1299, MDA-MB-231, U251, A549, and U2OS cells were kindly provided by Prof. Peng Jiang. LOVO, RKO, DLD-1, and CW2 cells were obtained from the Cell Resource Center, IBMS, CAMS/PUMC. T47D cell line was purchased from Servicebio. Caco-2 cell line was donated to Prof. Jin Zeng.

All the cells were cultured in a 5% CO_2_ humidified incubator (Thermo Fisher Scientific) at 37°C. MDA-MB-231, U87, U251, A549, DLD-1, T47D, and CW2 cells were routinely maintained in Dulbecco's modified Eagle's medium (Gibco, Cat# C11995500BT). U2OS and HCT116 cells were maintained in McCoy's 5A medium (VivaCell, Cat# C3020), RKO cells in MEM-ALPHA medium, and H1299 and Caco-2 cells in RPMI1640 medium (VivaCell, Cat# C3001). LOVO cells were cultured in F-12K medium (Gibco, Cat# 21127022). All media, if not specified, were supplemented with 10% fetal bovine serum (GEMINI, Cat# 900-108). All cells were incubated in culture medium without 1% penicillin/streptomycin (Thermo Fisher, Cat# 15140122) for <2 months and examined for mycoplasma annually.

### siRNAs

The siRNAs used in this study were synthesized by Gene Pharma, with sequences as follows: siCtrl: 5′-UUCUCCGAACGUGUCACGUTT-3′; siFOXO1 #1: 5′-GGAGGUAUGAGUCAGUAUATT-3′; siFOXO1 #2: 5′-GCCCUGGCUCUCACAGCAATT-3′; siFOXO1 #3: 5′-GUUCAUUCGUGUGCAGAAUTT-3′; and siG6PD: 5′-AAACCCACUCUCUUCAUCAGCUCGU-3′.

Cells were transfected with siRNAs (20 nM) using Lipofectamine RNAiMAX (Thermo Scientific, Cat# 13778075) transfection agent according to the manufacturer's instructions.

### Plasmids

The coding sequences corresponding to the full-length human FOXO1 gene were amplified by PCR from the cDNA library of 293T cells and then cloned into the PRK5-flag empty vector. The primers used for this cloning process were 5′-CGCGGATCCATGGCCGAGGCGCCTCA-3′ (forward) and 5′-ACGCGTCGACTCAGCCTGACACCCAGCTATGTGT-3′ (reverse).

The expression vectors for PRK5-GST-G6PD and pBabe-puro-G6PD were kindly provided by Prof. Peng Jiang. Retrovirus-stabilized cells were screened with 1 μg/ml puromycin (Yeasen, Cat# HB181128). The expression vector pCDNA-v5/Hs-FOXO1 was purchased from the Plasmid Resource Sharing Platform of Suzhou Research Institute of Biochemical Cells (Cat# SP-2267). Chemifect (FENGRBIO, Cat# FR-01) was used for cell transfection assays.

### Antibodies and reagents

Antibodies against the following proteins/epitopes were used for immunoblotting with the sources, catalog numbers, and dilutions indicated: Actin (EASYBIO, Cat# BE0021, RRID: AB_3107174, 1:5000), FOXO1 (Cell Signaling Technology, Cat# 2880S, 1:1000), and G6PD (Proteintech, Cat# 25413-1-AP, RRID: AB_2880066, 1:1000).

AS1842856 (Cat# S8222), DOX (Cat# E2516), and CHX (Cat# S7418) were purchased from Selleck. Crystal violet (Cat# 61135), 0.4% trypan blue solution (Cat# 93595), H_2_O_2_ (Cat# 1.08597), G6P (Cat# 10127647001), 6PG (Cat# P7877), and NADP^+^ (Cat# 481972) were purchased from Sigma–Aldrich.

### Data analysis on public datasets

The survival analysis of colorectal cancer and breast cancer based on FOXO1 (202724_s) or G6PD (202275) expression was performed using the online database Kaplan–Meier Plotter (Győrffy, 2024a, b).

### Collection of tumor tissues and quantitative detection

This study was approved by the Research Ethics Committee of General Hospital of Ningxia Medical University (KYLL-2022-1213). Ethical approval and informed consent were obtained from all participants. Colorectal cancer tissues, as well as adjacent normal tissues, were collected from patients at the General Hospital of Ningxia Medical University. The tumor tissue samples were procured during surgical procedures. Following RNA extraction using an RNA extraction kit (DAKEWE, Cat# 8034111), qRT-PCR was employed for quantitative detection of FOXO1 mRNA levels in these tissue samples, with actin serving as an internal control.

### Statistical analysis

All results are presented as the mean with standard deviation (SD) of at least triplicates. Bar graphs were generated using Graphpad Prism v8.0.1. Statistical significance was calculated by unpaired two-tailed *t*-test or as specified in figure legends (*P < 0.05, **P < 0.01, ***P < 0.001). The sample size (*n*) defines the number of mice for animal experiments and the number of independent biological experiments for cell or *in vitro* studies. All data points are displayed in the plots when possible.

## Supplementary Material

mjaf021_Supplemental_File

## Notes

**Author contributions:** conceptualization: L.L.; methodology: L.L., P.J., Y.W., X.Z., J.Z., and M.W.; investigation: L.L., X.Z., J.Z., M.W., M.Z., X.W., Y.H., D.Y., Z.W., and W.W.; visualization: X.Z., J.Z., M.W., and M.Z.; funding acquisition: L.L. and Y.W.; project administration: L.L.; supervision: L.L.; writing—original draft: L.L., X.Z., J.Z., and M.W.; writing—review & editing: L.L., Y.W., P.J., J.Z., and M.W.
