# Supplementary material for: FOXO1 enhances G6PD expression to promote cancer cell antioxidative capacity
Source: J Mol Cell Biol. 2025 Oct 22;17(5):mjaf021. doi: 10.1093/jmcb/mjaf021 (PMC12750447; doi:10.1093/jmcb/mjaf021)
Supplement: mjaf021_Supplemental_File [file mjaf021_supplemental_file.pdf]

Supplementary material

FOXO1 enhances G6PD expression to promote cancer cell antioxidative capacity

Xianhong Zhang[1,2,†], Jie Zhang[1,2,†], Mengmeng Wei[1,2,†], Min Zhao[1,2], Xiaoxiong Wang[3], Yongfeng Hui[3], Dongdong Yuan[1,2], Zijiao Wang[1,2], Wei Wu[1,2], Peng Jiang[4], Yujiong Wang[1,2,*], and Le Li[1,2,*]

[1] School of Life Sciences, Ningxia University, Yinchuan 750021, China

[2] Key Lab of Ministry of Education for Protection and Utilization of Special Biological Resources in Western China, Ningxia University, Yinchuan 750021, China

[3] General Hospital of Ningxia Medical University, Yinchuan 750021, China

[4] School of Life Sciences, Tsinghua University, Beijing 100084, China,

[†] These authors contributed equally to this work.

[*] Correspondence to: Le Li, E-mail: leli@nxu.edu.cn; Yujiong Wang, E-mail: wyj@nxu.edu.cn

Supplementary Materials and methods

Western blot analysis

Cells were collected and washed once with precooled PBS (Servicebio, Cat# G4202), and then centrifuged at 3000 rpm for 5 min to collect the pellet. The cells were lysed in 100 μl of RIPA buffer (10 mM Tris-HCl, pH 7.5, 5 mM EDTA, 150 mM NaCl, 1% NP-40, 1% sodium deoxycholate, and 0.025% SDS) containing protease and phosphatase inhibitors (Selleck, Cat# B14001 and Cat# B15001) to extract total protein. The lysed cells were subjected to non-contact ultrasonic disruption for 5 min, followed by centrifugation at 12000 rpm at 4 ℃ for 10 min. The supernatant was collected and protein concentrations were determined using the BCA assay (Sangon, Cat# C503021). Proteins were mixed with 5× loading buffer and heated at 95 ℃ for 10 min. Protein concentrations were adjusted using 1× loading buffer. The proteins were then separated by SDS-PAGE on a polyacrylamide gel consisted of 10 ml 10% resolving gel (comprising 4 ml H_2O, 3.3 ml 30% acrylamide, 2.5 ml 1.5 M Tris at pH 8.8, 0.1 ml 10%SDS, 0.1 ml 10%APS, and 0.004 ml TEMED) and 10 ml 5% stacking gel (comprising 6.8 ml H_2O, 1.7 ml 30% acrylamide, 1.25 ml 1 M Tris at pH 6.8, 0.1 ml 10%SDS, 0.1 ml 10%APS, and 0.01 ml TEMED). Subsequently, the proteins were transferred to a nitrocellulose membrane (Merck, Cat# HATF00010). The membrane was blocked with 5% non-fat milk (Sangon, Cat# A600669) in TBS/1% Tween for 1 h at room temperature and

then washed with TBS/1% Tween. The membranes were then incubated with primary antibodies overnight at 4 ℃. After washing with TBS/1% Tween, membranes were incubated with secondary HRP-conjugated antibodies for 1–2 h at room temperature. HRP signals were visualized using ECL (Tanon, Cat# 180). The bands were detected using a Tanon Imaging System (Tanon, China).

RNA-seq and data analysis

After transfection of HCT116 cells with control and three different siRNAs, total RNA was isolated from HCT116 cells by Trizol (Ambion). Total amounts and integrity of RNA were assessed using the RNA Nano 6000. Total RNA was used as input material for the RNA sample preparations. Briefly, mRNA was purified from total RNA by using poly-T oligo-attached magnetic beads. After insert size met the expectation, qRT-PCR was used to accurately quantify the effective concentration of the library (>2 nM) to ensure the quality of the library.

Reference genome and gene model annotation files were downloaded from genome website directly. Index of the reference genome was built using Hisat2 (v2.0.5) and paired-end clean reads were aligned to the reference genome using Hisat2 (v2.0.5). We selected Hisat2 as the mapping tool because Hisat2 can generate a database of splice junctions based on the gene model annotation file and thus a better mapping result than other non-splice mapping tools. ClusterProfiler R package (3.8.1) was used to test the statistical enrichment of differential expression genes in KEGG pathways.

Quantitative RT-PCR analysis

Total RNA was isolated from the cells using an RNA extraction kit (DAKEWE, Cat# 8034111). Then, the ReverAid First Strand cDNA Synthesis Kit (Thermo Scientific, Cat# K1622) was used to reverse-transcribe cDNA from RNA (1 μg). Subsequently, cDNA (0.2 μg) was used as a template and amplified by quantitative PCR using SYBR Green PCR Master Mix (Genestar, Cat# A308).

The primer sequences used for different genes were as follows: FOXO1, 5′-CCAGCCCAAACTACCAAAAATA-3′ and 5′-GAGGAGAGTCAGAAGTCAGCAAC-3′; G6PD, 5′-TGGAGAATGAGAGGTGGGA-3′ and 5′-TGCTGGTGGAAGATGTCG-3′; β-actin, 5′-GTCTTCCCCTCCATCGTG-3′ and 5′-AGGGTGAGGATGCCTCTCTT-3′.

Chromatin immunoprecipitation (ChIP) and luciferase reporter assays

We used JASPAR1 (http://jaspar.genereg.net) to identify potential FOXO1 response elements in G6PD genes. For ChIP assays, cells were cross-linked using 1% formaldehyde for 15 min at room temperature, and cross-linking was stopped by adding 125 nM glycine (final concentration). Cell lysates (1% SDS, 10 mM EDTA, and 50 mM Tris-Cl, pH 8.1) were sonicated to generate DNA fragments with an average size of <1000 bp and then immunoprecipitated with the indicated

antibodies. The bound DNA fragments were eluted and amplified using PCR. The primer sequences used were as follows: RE, 5’-CCGCTCGAGCCTTCCTCTCCAACTGTGCTC-3’ and 5’-CCCAAGCTTTCTGTTCACCAAACTTGACTG-3’; Actin, 5’-TGCTATCCCTGTACGCCTCT-3’ and 5’-CTCCTTAATGTCACGCACGA-3’.

For qChIP experiments, 2× RealStar Power SYBR qPCR Mix (Genestar, Cat# A308) was used to detect enrichment status.

The wild-type FOXO1 binding region (RE2) with the sequence of AGAAACAGTATGA and the mutant FOXO1 binding region (RE2) with the mutated nucleotides underlined (sequence: AG<u>CTCTCC</u>TATGA) were cloned into the pGL3-basic vector (Promega, Cat# E1751). Luciferase activity was determined using a dual-luciferase assay system (Promega, Cat# E1910). Transfection efficiency was normalized to the Renilla luciferase activity.

G6PD enzyme activity assays

The combined activity of G6PD and 6-phosphogluconate dehydrogenase (6PGD), the second enzyme of PPP that produces NADPH, was determined by the rate of conversion of $NADP^+$ to NADPH in the presence of glucose-6-phosphate (G6P). The 6PGD activity was measured by the conversion of $NADP^+$ to NADPH in the presence of 6-phosphogluconate (6PG). G6PD activity was calculated by subtracting 6PGD from the combined activity. The cells were used to generate cell lysates. The reaction buffer, containing 50 mM Tris at pH 8.1, 1 mM $MgCl_2$, 200 μM G6P (Sigma, Cat#V900924), 200 μM 6PG (Sigma, Cat#P7877), and 100 μM $NADP^+$ (Sigma, Cat#N5755), was added to the cell lysate. The protein concentration was quantified and analyzed using the BCA Protein Assay Kit (Sangon, Cat# C503021). The absorption peak of the mixed solution at 340 nm was detected using a microplate reader.

Measurement of NADPH and ROS levels

NADPH levels and $NADP^+$/NADPH ratios were determined using an $NADP^+$/NADPH quantification kit (BioVision, Cat# K347).

The ROS levels were determined as follows: The cells were incubated at 37 ℃ for 30 min in 1× PBS containing 10 μM 2′,7′-dichlorodihydrofluorescein diacetate (DCFH-DA; Selleck, Cat# S9687). The cells were then washed twice with 1× PBS, treated with trypsin, and resuspended in 1× PBS. Fluorescence was measured immediately using a flow cytometer (Wellgrow, China).

Apoptosis and cell survival assays

Cell apoptosis was analyzed using an Annexin V-FITC/PI Apoptosis Detection Kit (Solarbio, Cat# CA1020). The cultured cells were treated with trypsin and washed twice with cold 1× PBS. The

supernatant was discarded and the pellet was resuspended in 1× binding buffer to form a cell suspension. Subsequently, the cell suspension (100 μl) was transferred to a culture tube and incubated with Annexin V-FITC (5 μl)/PI (5 μl) for 15 min at room temperature in the dark. Binding buffer (900 μl) was added to the cell samples, and the samples were analyzed using a flow cytometer.

Cells were transfected with siRNA for 24 h and then seeded in 6-well plates in triplicates at a density of 1×10^5 cells per well. Cells were treated with 100 μM H_2O_2 after fixing on the plate and then incubated for 24 h. For trypan blue staining, cells were harvested with trypsin, washed once, and resuspended in 1× PBS, and then trypan blue was added to the cell suspension at a volume ratio of 9:1. The live cell ratio was determined by counting the cells using a hemocytometer.

Cell proliferation assay and soft agar assays

Cells were transfected with siRNAs for 48 h and then seeded in 6-well cell culture dishes at a density of 20000 cells/well. Subsequently, the cells were supplemented with or without 100 μM H_2O_2. The cell number at a specified time point was recorded using a hemocytometer. For the crystal violet (CV) staining assay, the cells were fixed with 4% polyformaldehyde for 10 min and then stained with 0.05% CV for 10 min. After that, the cells were washed with distilled water and imaged.

Cells were transfected with FOXO1 siRNA for 24 h. Then, the transfected cells were supplemented with or without 100 μM H_2O_2 and mixed with 20% fetal bovine serum (FBS) containing 0.3% agarose. The cell-agarose mixture was plated on a 0.6% agarose base in 12-well plates (2000 cells per well). The cells were cultured at 37 ℃ in a 5% CO_2 incubator. After 2–3 weeks, the colonies were stained with 0.05% crystal violet in 1× PBS for 1 h. Once the colonies turned blue, they were counted under a microscope and photographed.

Metabolite extraction and mass spectrometry (MS)-based metabolomics analysis.

Cells were transfected with pBabe-puro-G6PD or knockdown FOXO1 use siRNA, collected in cold 1× PBS using cell scrapers, and processed using a high-speed refrigerated centrifuge at 12000 rpm for 10 min. The supernatant was removed, and the sediment was quickly frozen using liquid nitrogen. Finally, the samples were stored for subsequent metabolomic analyses.

The cell residue was treated with a precooled methanol/acetonitrile/water (*v*/*v*, 2:2:1) mixed solution (1 ml) and ultrasonicated in an ice bath for 1 h, after which the metabolites were extracted. The mixture was incubated at −20 ℃ for 1 h and spun in a high-speed refrigerated centrifuge at 14000 rpm for 20 min. The samples were then transferred to a sample bottle for LC/MS analysis.

The LC/MS portion of the platform was based on the Shimadzu Nexera X2 LC-30AD system equipped with an ACQUITY UPLC BEH Amide column (1.7 μm, 2.1 mm × 100 mm, Waters) and a triple quadruple mass spectrometer (5500 QTRAP, AB SCIEX). Metabolites were detected in

electrospray negative-ionization and positive-ionization modes. Approximately 2 μl of samples were injected sequentially with an LC autosampler. The ACQUITY UPLC BEH Amide column (1.7 μm, 2.1 mm × 100 mm, Waters) was heated to 45 ℃ at a flow rate of 300 μl/min. The gradient method was used to separate and purify the samples. Raw MRM data were processed and analyzed using the MultiQuant software.

Raw data were normalized using a statistically significant threshold of fold change (FC) and two-tailed Student's *t*-test (*P*-value) to discriminate metabolites. FC was evaluated as the logarithm of the average mass response (area) ratio between two arbitrary classes. The *P*-value was calculated using one-way analysis of variance for multiple-group analysis. When the FC was >1.5 and the *P*-value was <0.05, the metabolites were considered statistically significant. The identified differential metabolites were analyzed using cluster analysis using R package.

Supplementary Figures

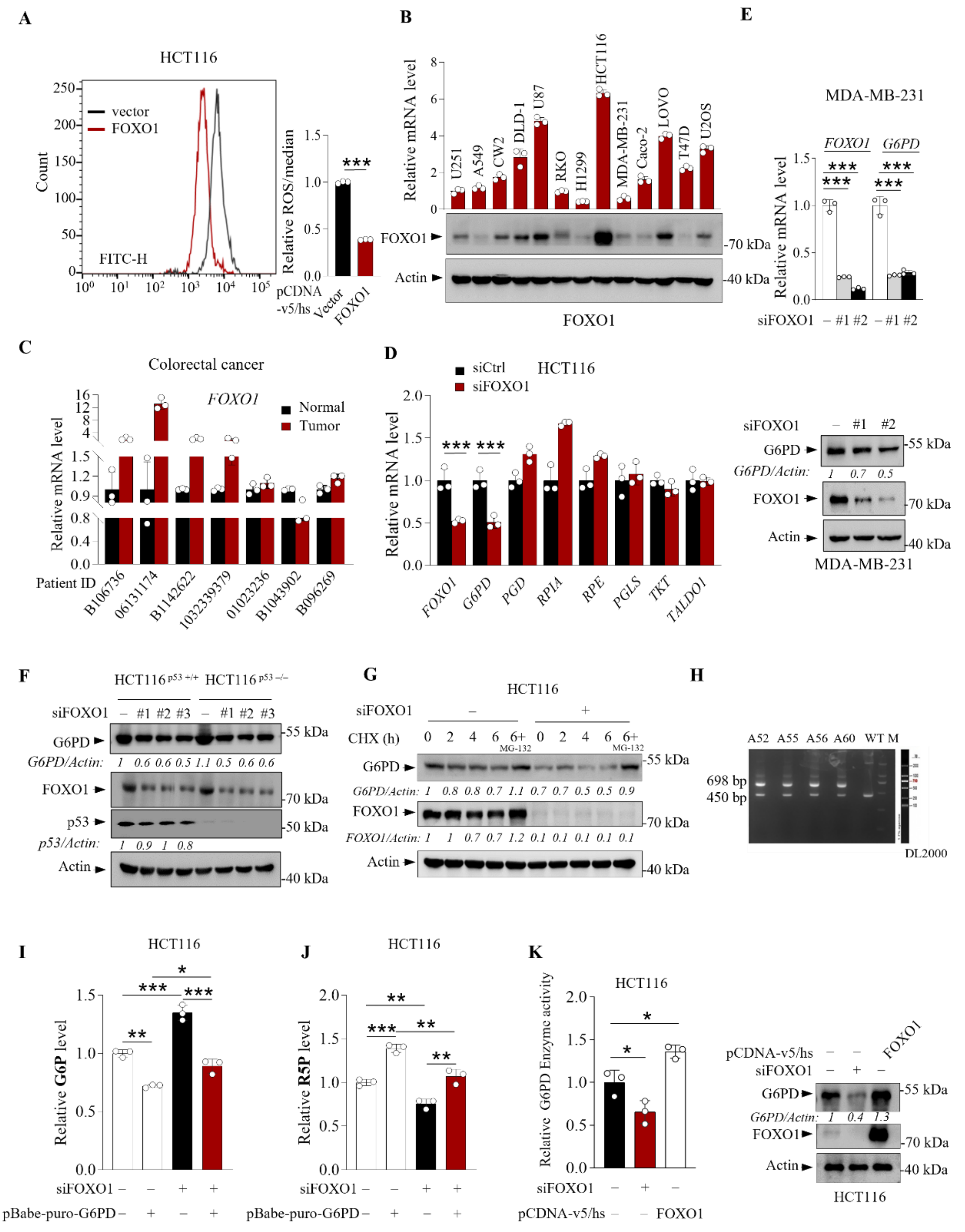

Supplementary Figure S1. FOXO1 regulates the expression of G6PD. (**A**) The ROS level in HCT116 cells transfected with pCDNA-v5/hs-FOXO1 or pCDNA-v5/hs-vector is shown. Cells were incubated at 37 ℃ for 30 min in 1× PBS containing 10 μM DCFH-DA under dark conditions. ROS levels were determined by flow cytometry, and the results were presented as mean fluorescence

intensity values (±SD) in the form of histograms. (**B**) FOXO1 mRNA and protein levels in U251, A549, CW2, DLD-1, U87, RKO, H1299, HCT116, MDA-MB-231, Caco2, LOVO, T47D, and U2OS cells were analyzed by qRT-PCR (top, n = 3) and western blotting (bottom). (**C**) Relative FOXO1 mRNA levels in human Colorectal cancer tissues and adjacent tissues are shown (n = 7 patients). (**D**) HCT116 cells were transfected with control siRNA or FOXO1 siRNA for 48 h. *FOXO1, G6PD, PGD, RPIA, RPE, PGLS, TKT, TALDO1* mRNA levels were analyzed by qRT-PCR (n = 3). (**E**) MDA-MB-231 were treated with control or two independent siRNAs of FOXO1. mRNA and protein levels were detected by qRT-PCR (top, n = 3) and western blotting (bottom), respectively. (**F**) HCT116$^{p53+/+}$ and HCT116 $^{p53-/-}$ were treated with control siRNA or FOXO1 siRNA for 24 h. p53, G6PD and FOXO1 protein levels were analyzed by western blotting. (**G**) HCT116 cells were transfected with control siRNA or FOXO1 siRNA, followed by treatment with 100 mg/ml CHX for different times. Additionally, cells were exposed to MG132 (15 μM) for 6 h, starting 24 h post-transfection. G6PD and FOXO1 protein levels were analyzed by western blotting. (**H**) Identification of FOXO1-floxed mice. A52, A55, A56, A60: heterozygote (FOXO1$^{-/+}$); WT: wild-type; M: DL2000 DNA marker from Transgen. Wild-type: 450 bp. Heterozygote (FOXO1$^{-/+}$): 450 bp and 698 bp. (**I** and **J**) HCT116 cells overexpressing pBabe-puro-G6PD or vector control in the presence or absence of FOXO1 siRNA. G6P and R5P levels were detected by LC-MS (n = 3). (**K**) HCT116 cells were transfected with control, FOXO1 siRNA or pCDNA-v5/hs-FOXO1. Then, G6PD activity was detected as shown (n = 3), and G6PD and FOXO1 protein levels were analyzed by western blotting. For all graphs, data are presented as mean ± SD, *$P < 0.05$, **$P < 0.01$, ***$P < 0.001$. Statistical analysis was performed with unpaired two-tailed t-test.

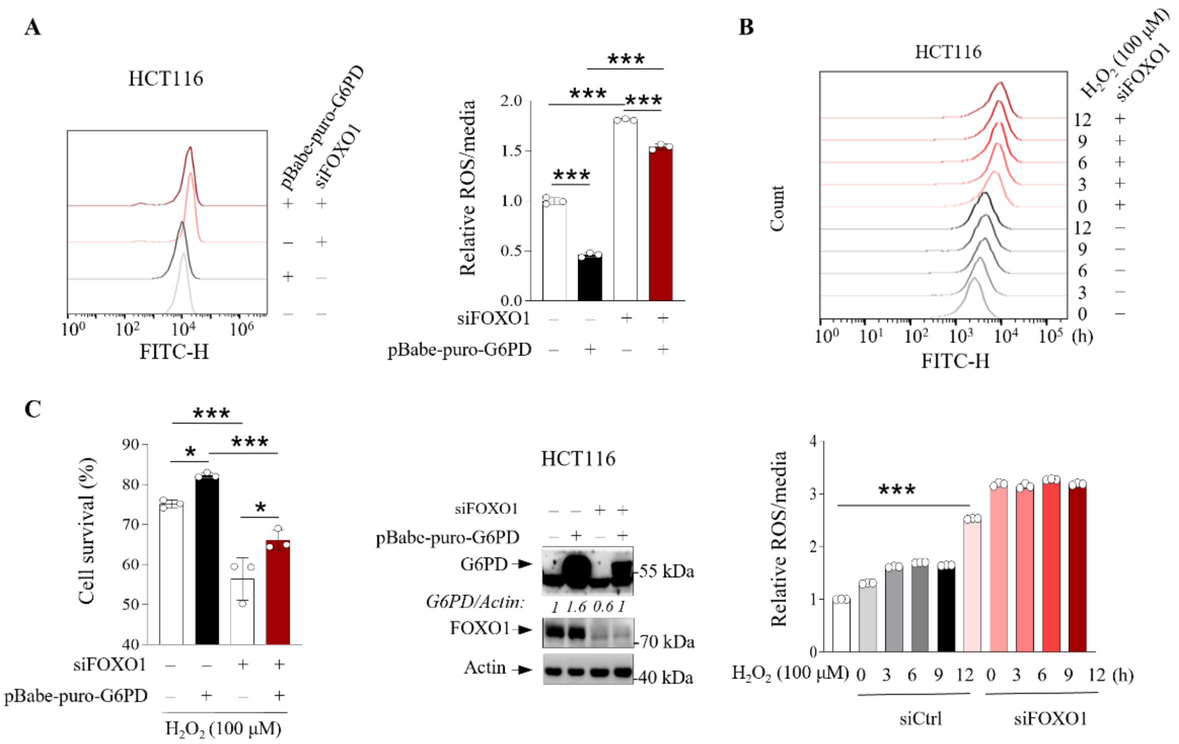

Supplementary Figure S2. FOXO1 activates G6PD expression to enhance cellular antioxidant capacity. (**A**) ROS level in HCT116 cells overexpressing pBabe-puro-G6PD or Vector in the presence or absence of FOXO1 siRNA is shown. Cells were incubated at 37 ℃ for 30 min in 1 × PBS containing 10 μM DCFH-DA under dark conditions. ROS levels were determined by flow cytometry, and the results were presented as mean fluorescence intensity values (±SD) in the form of histograms. G6PD and FOXO1 protein levels were analyzed by western blotting. (**B**) The ROS levels (top) of HCT116 cells were detected by adding 100 μM H_2O_2 at different times in the presence or absence of FOXO1 siRNA. After 24 h of siRNA treatment, cells were treated with H_2O_2. Quantification is represented as a bar chart (bottom). (**C**) HCT116 cells were transfected with pcDNA-V5/hs-FOXO1 or vector control and then treated with 100 μM H_2O_2 for 24 h, beginning 24 h post-transfection. Cell survival was assayed by crystal violet staining (left). G6PD and FOXO1 protein levels were analyzed by western blotting (right). For all graphs, data are presented as mean ± SD, $^{*}P < 0.05$, $^{**}P < 0.01$, $^{***}P < 0.001$. Statistical analysis was performed with unpaired two-tailed *t*-test (**B**) or two-way ANOVA followed by Tukey's multiple-comparison test (**A**, **C**).

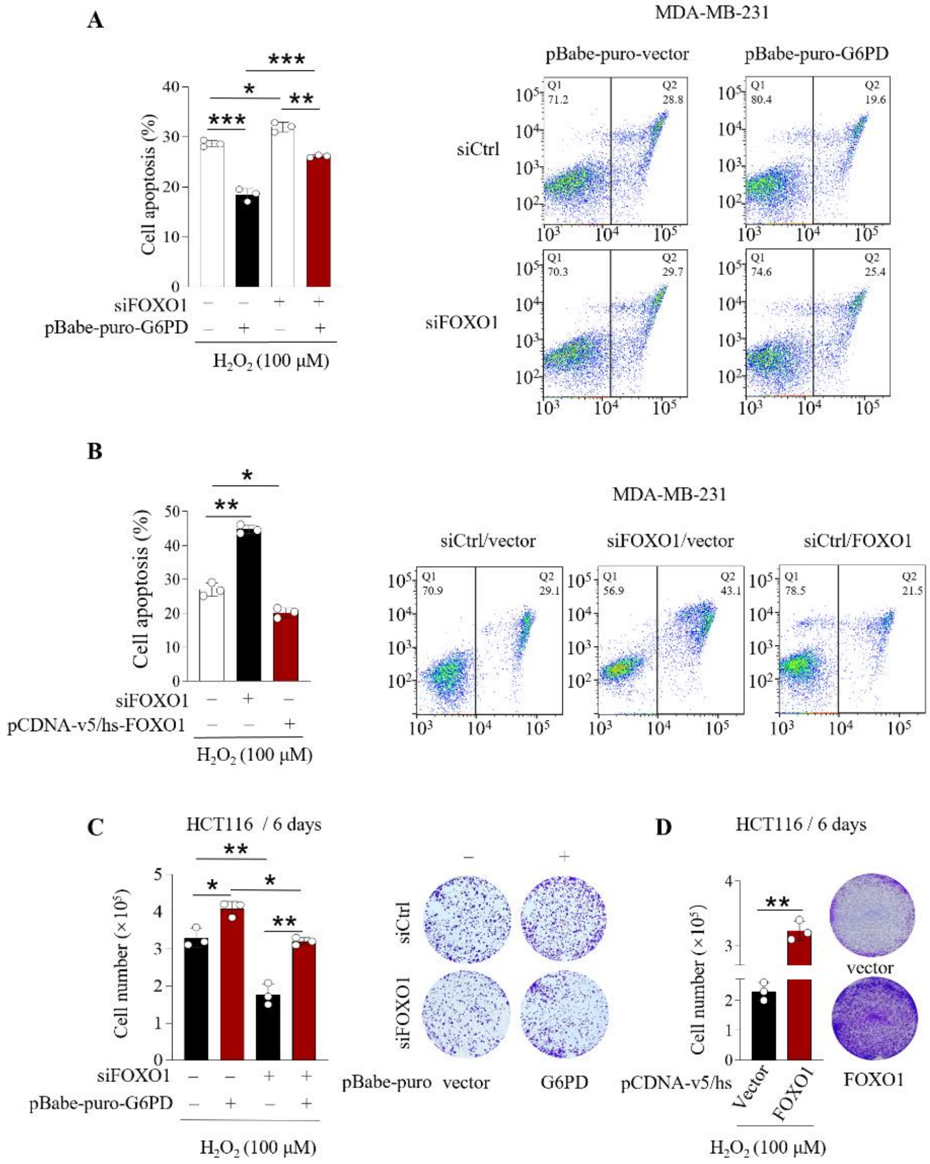

Supplementary Figure S3. G6PD is involved in FOXO1-mediated apoptosis under oxidative stress. (**A**) MDA-MB-231 cells stably overexpressing G6PD or the vector control in the presence or absence of FOXO1 siRNA were treated with 100 μM H_2O_2 for 24 h. Cell death was analyzed using flow cytometry after PI staining. (**B**) MDA-MB-231 cells overexpressing FOXO1 or the vector control were treated with 100 μM H_2O_2 for 24 h. Cell death was analyzed using flow cytometry after PI staining. (**C**) CT116 cells overexpressing G6PD or vector control in the presence or absence of FOXO1 were treated with 100 μM H_2O_2. Cell proliferation was analyzed by crystal violet staining. Representative images of cells stained with crystal violet on Day 6. (**D**) HCT116 cells overexpressing FOXO1 or vector control were treated with 100 μM H_2O_2. Cell proliferation was analyzed by crystal violet staining. Representative images of cells stained with crystal violet on Day 6. For all graphs, data are presented as mean ± SD, $*P < 0.05$, $**P < 0.01$, $***P < 0.001$. Statistical analysis was performed with unpaired two-tailed *t*-test (**D**) or two-way ANOVA followed by Tukey's multiple-comparison test (**A**–**C**).

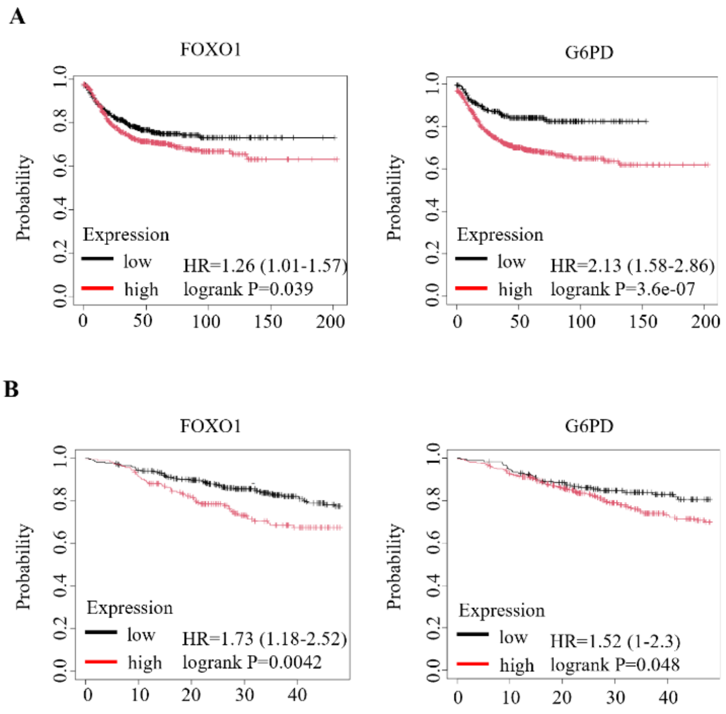

Supplementary Figure S4. Kaplan-Meier survival analysis of the gene signature with colorectal cancer (**A**) and breast cancer (GSE25066) (**B**) based on FOXO1 or G6PD expression.
